# Ultraprocessed Foods in Popular Children’s Television Series

**DOI:** 10.5888/pcd23.250301

**Published:** 2026-04-02

**Authors:** Milia Tzoutzou, Paraskevi Detopoulou, Antonia-Leda Matalas, Eirini Bathrellou

**Affiliations:** 1 Department of Nutrition and Dietetics, School of Health Sciences and Education, Harokopio University of Athens, Greece; 2 Department of Nutritional Science and Dietetics, University of the Peloponnese, Messinia, Greece

## Abstract

**Introduction:**

Research on children’s television programming examining the quality of presented foods finds a prevalence of processed foods, though the degree of processing of the foods shown has not been extensively examined. This study aims to assess the quality of the food presented in popular children’s television series, based on the NOVA food classification system, and distinguish between the way foods are presented.

**Methods:**

We selected 100 episodes of 10 cartoon television series that had large audiences of children. Food items, either eaten by heroes or introduced visually or verbally in television series, were recorded per episode. They were then classified according to the NOVA system. Differences by the series continent of origin (Europe, North America, or Asia) were also examined.

**Results:**

The median number of food recordings per episode was 3.0, with most (61.5%) classified as foods introduced visually or verbally. More than half of all foods featured per episode were ultraprocessed foods (UPF) and one third of them were minimally processed foods (MPF), while other NOVA food categories rarely appeared. Both UPF and MPF were presented more often as foods introduced visually or verbally rather than as foods eaten, but UPF represented 72% of the foods eaten per episode. European cartoons broadcasted more UPF items compared with North American television series.

**Conclusion:**

UPF dominated in children’s television series, especially those of European origin, raising a public health concern for Europe.

**Summary**

**What is already known about this topic?**

Processed foods are prevalent in children’s television programming. The degree of their processing according to the NOVA system has not been extensively examined.

**What is added by this report?**

We examined food-related content in popular children’s media, considering the degree of food processing and geographical origin. Ultraprocessed foods (UPF) were the most abundant foods in broadcasts; European cartoons had a greater number of UPF compared with North American cartoons.

**What are the implications for public health practice?**

Television series can negatively influence children’s dietary choices. Public health programs in cooperation with media producers can use these findings to create healthier media representations and promote children’s well-being.

## Introduction

Research on children’s television programming and children’s movies examining the quality of presented foods finds a prevalence of processed foods. For example, sugar-sweetened beverages and unhealthy snacks are notably common in children’s movies (1) and adolescents’ television shows (2), while another study indicates that nearly twice the amount of airtime in children’s television programming is devoted to showcasing unhealthy foods with high sugar and processed fat content (3). By considering visual, verbal, or actual eating scenes within children’s animated programs it has been noted that sweets, salty snacks, and sugar-sweetened beverages accounted for 42% of all representations (4). Our research team has also found that convenience food items, such as sweets and salty snacks, prevail in terms of actual consumption in popular children’s programming (5,6).

Several classifications of broadcast foods relating to healthiness or unhealthiness have been used in earlier publications. Foods have been categorized in national food recommendations as nutritious or nonnutritious (7); healthy, unhealthy, or neutral (1); or healthy, mixed, and unhealthy (8). Some studies provide a food-centered categorization of the items (ie, dairy, beverages, snacks, desserts) (5,9,10). Olafsdottir and Berg (11,12) have classified foods as “high calorie–low nutrient” and “fruits and vegetables.” During recent decades, another parameter that has been gaining popularity as an indicator of the healthiness or unhealthiness of food is the degree of processing, in terms of nature, extent, and purpose, which is described in the NOVA classification system (13). Ultraprocessed foods (UPF) encompass ready-to-eat as well as ready-to-heat foods, produced through industrial methods. Some examples of UPF are cereal bars, savory snacks, processed meats, prepackaged frozen meals, soft drinks, sweetened beverages, instant soups, and sauces (14). UPF consumption has raised concern regarding its association with impaired health outcomes, such as obesity and other cardiometabolic disease risk factors (15). This link may be partially explained by the fact that they are palatable and “comfort” foods but are also calorie-dense, high in fat, and low in dietary fiber (16). The degree of processing of foods shown in cartoons has not been extensively examined by previous research; Horta et al (17) use the widely accepted, international classification of the NOVA food system to categorize foods. Moreover, because UPF consumption varies among countries (18) and because cartoons may be seen as a reflection of the cultures and everyday life habits of a place, it would be interesting to investigate the food-related content of children’s television programming in relation to its geographical origin, an aspect that has been rarely studied (19,20).

The objective of our study was to classify all food items presented in popular children’s television series in Greece according to the NOVA system. Moreover, as food marketing employs both explicit and implicit messages to influence children’s behavior (21), distinguishing between the way the foods are presented may be of interest. We aimed to distinguish between foods shown to be ingested on screen and those that were presented solely verbally or visually. We also examined relevant disparities pertaining to the cartoon’s geographic origin.

## Methods

### Selection of television series

We selected 100 episodes of the 10 most popular television series, based on audience ratings in Greece, presented to children (aged 4 to 14 years). On request, the Nielsen Audience Company submitted data showing broadcasting ratings for the months of October 2011 and June 2012. The audience ratings were based on the Average Minute Rating (AMR) scale, which measures the percentage of the population in the target group watching a program during the average minute of a given period. Out of the 84 broadcasted television series, 22 had an AMR rating exceeding 7%. Among the latter, our study focused on the top 10 series (all cartoons), having an AMR of 9.5% or higher, which were *Ben 10*, *Dora the Explorer*, *Jewelpet*, *Lazy Town*, *Penguins of Madagascar*, *PitchiePitchie Pitch*, *SpongeBob SquarePants*, *Teen Days*, *Tom and Jerry*, and *Tutenstein.*

The selected television series were classified according to the country of production, then the countries were grouped into their respective continents. Three production continents were identified:

1. Asia: *Jewelpet* and *PitchiePitchie Pitch* (Japan).
2. North America: *Ben 10*, *Dora the Explorer*, *Penguins of Madagascar*, *SpongeBob SquarePants*, *Tom and Jerry*, and *Tutenstein.*
3. Europe: *Teen Days* (Italy) and *Lazy Town* (Ireland).

### Classification of foods featured in the episodes

#### Food classification by presentation

All food items shown in the television series were recorded. Whenever a cartoon character (whether primary or secondary, human or animal) mentioned or ate a food or a drink, this item was recorded. All noningested foods and actual eating situations were recorded, irrespective of whether they were integral to a central scene or existed in the surroundings. Two independent raters with a background in nutrition science viewed all 100 episodes. In cases where a food item was eaten by a character, the food was classified as “foods eaten,” while foods and drinks presented on screen or verbally discussed among the cartoon characters were classified as “foods introduced visually or verbally.” Foods presented visually referred to food items that were depicted on screen (eg, an apple, a cake, a bowl with corn flakes being on the table during breakfast time). Foods presented verbally were those for which any kind of food-related aspect was expressed by cartoon heroes; for example, some sensorial properties (ie, smell, taste, texture, color, shape), or implications by the heroes about their consumption or nutritional value.

Recording and counting of the food items was based on the assumption that for every food item featured in any way, 1 item was recorded. For multiple food items featured, each one of them was counted separately. For instance, if the raters observed a cartoon character eating an apple in an episode’s scene, they recorded 1 item of apple. If a plate with various food items such as chicken, rice, and lettuce was portrayed, then 3 items were counted. Eating occasions with an extreme consumption were not included in the analysis (ie, one case of a character binge-eating). Other foods excluded from recording were those where the food could not be specified.

#### Food classification using the NOVA system

Foods reviewed were classified according to the NOVA food system (14)*.* The NOVA food classification system categorizes foods into 4 groups based on the level of processing: unprocessed or minimally processed foods (MPF), processed culinary ingredients (PCI), processed foods (PF), and UPF. Foods in these categories can be exemplified as follows: MPF includes fruits, vegetables, nuts, and eggs; PCI includes olive oil, sugar, honey, and butter; PF includes tomato paste, freshly made cheeses, and canned fish; and UPF includes fast foods, soft drinks, processed breads, biscuits, breakfast cereals, and sweetened yogurts (14). Classification of food items according to the NOVA system was done by the 2 raters who recorded the foods featured, with high degree of agreement between them. In case of disagreement, a third researcher was involved, and a group discussion was carried out. It should be noted, however, that as it is difficult to distinguish between homemade and industrially made foods projected in cartoon series, raters agreed to a priori categorize certain kinds of foods (eg, cakes, cereal bars, hot dogs, burgers, salty snacks) as UPF foods (13) unless otherwise stated in the episode.

### Statistical analysis

Descriptive statistics include the frequencies (absolute numbers) and percentages of foods eaten, foods introduced visually or verbally, and all foods featured per episode for all NOVA categories (ie, MPF, PCI, PF, and UPF). As the variables investigated were nonnormally distributed (Kolmogorov-Smirnoff criterion), values are presented as medians and interquartile ranges (Q2 [Q1–Q3], where Q2 = median; Q1 = 25th percentile; Q3 = 75th percentile), and nonparametric tests were applied for statistical comparisons. The Mann–Whitney and Kruskal–Wallis tests were used to test the differences between 2 or more categories, respectively. For post hoc analysis we used the Dunn test, in cases where a significant main effect was found. We used Bonferroni adjustment for multiple comparisons. Statistical significance for all tests was defined at α = .05. We used Stata version 17 (Stata Corp LP) software for all statistical analyses.

## Results

The food items featured in the episodes analyzed are listed in the Box, as classified according to the NOVA system, and Table 1 presents the frequencies of foods of each NOVA category per episode. Across 100 episodes, 457 foods were shown, with a median number recorded per episode of 3.0 (1.6–6.0). Among them, 176 (38.5%) were categorized as foods eaten, and the remaining 281 (61.5%) were classified as foods introduced visually or verbally. In 40 episodes, no MPF items were featured, while the number of episodes with no recordings of UPF items was 30. PCI and PF items were rarely featured. In all episodes reviewed, foods characterized as PCI were presented 5 times, of which 2 were shown to be eaten (olive and peanut butter). Foods characterized as PF items were presented 24 times, of which 9 concerned actual consumption (wine, canned fruits, and fish) and 15 visual presentations (eg, wine, cheese, pickles) (Table 1). While both UPF and MPF items were presented more often as foods introduced visually or verbally rather than as foods eaten (161 vs 117 of 278 recordings, and 102 vs 48 of 150 recordings) (Table 1), within the foods eaten per episode, 72.0% (33.3%–100%) were UPF, a median percentage that was significantly high compared with that of the other NOVA categories (Table 2). Moreover, the percentage of foods introduced visually or verbally or the percentage of all foods featured per episode did not differ between foods in the UPF and MPF categories (Table 2).

**Box. Foods and Beverages Presented in Television Series, by NOVA Food System Categories**

**Minimally Processed foods**

Tomato, carrot, lettuce, coconut, apple, vegetables, sugarcane, eggs, banana, nuts, fruit, watermelon, corn, papaya, orange, kiwi, apple, apple juice, pear, meat, ground meat, chicken, fish, turkey, seafood, onion, cherries, milk, grapefruit, ravioli, beans, coffee, tea, fish

**Processed Culinary Ingredients**

Olive, peanut butter, pickles

**Processed Foods**

Wine, cheese, canned fruits, canned fish

**Ultraprocessed Foods**

Biscuit, ice cream parfait, candies, cotton candy, chocolate smoothie, croissant, bread, salty snack, biscuit, birthday cake, ice cream, soda, sandwich, sausage, pizza, lollies, sweets, pancakes, cake, bread, hot dog, pizza, sandwich, ketchup, hamburger, popcorn, candies, hot chocolate, corn flakes

**Table 1:** Foods Featured in 100 Television Episodes of Popular Children’s Television Series,^a^ Analyzed by NOVA Category^b^

Classification	No. (%)				
	Minimally processed foods	Processed culinary ingredients	Processed foods	Ultraprocessed foods	Total
Foods eaten	48 (27.3)	2 (1.1)	9 (5.1)	117 (66.5)	176 (100)
Foods introduced visually or verbally	102 (36.3)	3 (1.1)	15 (5.3)	161 (57.3)	281 (100)
All foods featured	150 (32.8)	5 (1.1)	24 (5.3)	278 (60.8)	457 (100)

a Episodes broadcast in October 2011 and June 2012 were from the series *Ben 10*, *Dora the Explorer*, *Jewelpet*, *Lazy Town*, *Penguins of Madagascar*, *PitchiePitchie Pitch*, *SpongeBob SquarePants*, *Teen Days*, *Tom and Jerry*, and *Tutenstein*.

b Categories described in Monteiro et al (13).

**Table 2:** Differences in the Classification of Foods, by NOVA Food Category,^a^ Among Foods Presented Per Episode of Popular Children’s Television Series^b^

Classification	Percentage of items per episode^c^				
	Minimally processed foods	Processed culinary ingredients	Processed foods	Ultra-processed foods	*P* value
Foods eaten	0.0 (0.0–50.0)^z^	0.0 (0.0–0.0)^y^	0.0 (0.0–0.0)^y^	72.0 (33.3–100)^x^	<.001
Foods introduced visually or verbally	33.3 (0.0–66.6)^z^	0.0 (0.0–0.0)^y^	0.0 (0.0–0.0)^y^	55.5 (0.0–100)^z^	<.001
All foods featured	30.3 (0.0–61.9)^z^	0.0 (0.0–0.0)^y^	0.0 (0.0–0.0)^y^	58.5 (25.5–97.7)^z^	<.001

a Categories described in Monteiro et al (13).

b Episodes broadcast in October 2011 and June 2012 were from the series *Ben 10*, *Dora the Explorer*, *Jewelpet*, *Lazy Town*, *Penguins of Madagascar*, *PitchiePitchie Pitch*, *SpongeBob SquarePants*, *Teen Days*, *Tom and Jerry*, and *Tutenstein*.

c Percentages are for median values and interquartile ranges (Q2 = median; Q1 = 25th percentile; Q3 = 75th percentile). The Mann–Whitney test was used to test the differences between categories. Post hoc analysis was performed by using the Dunn test in cases where a significant main effect was found. Different letters (x, y, and z) denote significant differences in variables.

Table 3 presents the differences between continents in the percentage of the foods in a NOVA food category that were presented in different ways (eaten, introduced visually or verbally, and all foods shown), per episode (Appendix). A significant difference was reached only for all UPF items featured and the UPF items introduced visually or verbally. Pairwise comparisons showed that European series broadcasted more UPF items (total UPF items and UPF items introduced visually or verbally) per episode compared with Northern American ones. There were no significant differences for MPF or PF items between the regions.

**Table 3:** Differences in the Classification of Foods, by NOVA Food Category^a^, Per Episode of Popular Children’s Television Series^b^, by Series Continent of Origin^c^

Category	Percentage of items per episode^d^			
	Asia	Europe	North America	*P* value
**Minimally processed foods**				
Foods eaten	0.0 (0.0–100)	0.0 (0.0–50.0)	0.0 (0.0–58.3)	.72
Introduced visually or verbally	55.5 (0.0–100)	22.5 (0.0–44.6)	33.3 (0.0–100)	.12
All foods featured	36.6 (0.0–64.3)	20.0 (11.1–33.3)	33.3 (0.0–66.6)	.35
**Processed culinary ingredients**				
Foods eaten	0.0 (0.0–0.0)	0.0 (0.0–0.0)	0.0 (0.0–0.0)	.34
Introduced visually or verbally	0.0 (0.0–0.0)	0.0 (0.0–0.0)	0.0 (0.0–0.0)	.76
All foods featured	0.0 (0.0–0.0)	0.0 (0.0–0.0)	0.0 (0.0–0.0)	.48
**Processed foods**				
Foods eaten	0.0 (0.0–50.0)	0.0 (0.0–0.0)	0.0 (0.0–0.0)	.08
Introduced visually or verbally	0.0 (0.0–0.0)	0.0 (0.0–0.0)	0.0 (0.0–0.0)	.07
All foods featured	0.0 (0.0–8.3)	0.0 (0.0–0.0)	0.0 (0.0–0.0)	.11
**Ultraprocessed foods**				
Foods eaten	50.0 (0.0–100)	100 (50.0–100)	71.4 (33.3–100)	.62
Introduced visually or verbally	45.0 (0.0–100)	77.5 (55.3–100)^e^	50.0 (0.0–100)	.03
All foods featured	50.0 (0.0–100)	80.0 (66.6–88.8)^f^	50.0 (0.0–100)	.04

a Categories described in Monteiro et al (13).

b Episodes broadcast in October 2011 and June 2012 were from the series *Ben 10*, *Dora the Explorer*, *Jewelpet*, *Lazy Town*, *Penguins of Madagascar*, *PitchiePitchie Pitch*, *SpongeBob SquarePants*, *Teen Days*, *Tom and Jerry*, and *Tutenstein*.

c For each continent of origin, and within each NOVA food category, the value in the cell is the percentage of that NOVA food category to the total number of foods eaten or introduced visually or verbally or totally featured, respectively. For example, the value 80.0 for the ultraprocessed foods featured totally in Europe, means that 80.0% of all foods featured in European series were ultraprocessed foods.

d Percentages are for median values and interquartile ranges (Q2 = median; Q1 = 25th percentile; Q3 = 75th percentile). The Kruskal–Wallis test was used to test the differences between categories. Post hoc analysis was performed by using the Dunn test, in cases where a significant main effect was found. Bonferroni adjustment was performed for multiple comparisons. Pairwise comparisons were applied only when a significant difference was detected between groups.

e Significant pairwise comparisons, Europe vs North America, *P* = .03.

f Significant pairwise comparisons, Europe vs North America:* P* = .04.

## Discussion

We found that UPF were the most abundant foods broadcasted in all the episodes analyzed, while they constituted the most frequent NOVA category presented to be eaten in a typical episode. Only MPF were highly present in the television series studied, with a similar percentage of foods introduced visually or verbally in an episode compared with UPF in an episode. Additionally, European cartoons broadcasted more UPF items compared with North American cartoons.

In children’s television programming, research has shown that unhealthy foods are prominently featured (3,5,20). The food content we found herein pertains to UPF. However, the NOVA food system is not widely used as a medium for food classification in this context. Nevertheless, our findings align with those of other researchers using different food classification methods who also identified processed foods, such as sweets, as the second most common type of food depicted in children’s programming (20). In the meantime, an analysis of the nutritional content of foods featured on US television programs indicated that the total airtime for unhealthy food depictions was nearly twice as much as that for healthy food (3), while a study examining food consumption and gender disparities in popular television series found twice as many noncore foods (eg, sweets) consumed compared to core foods (eg, fruits) (22).

These depictions have raised concern in regard to their potential influence on children’s food behavior. A systematic review and meta-analysis on this issue found that in experimental studies children consumed more unhealthy calories than healthy ones after being exposed to unhealthy food marketing, with younger children being more susceptible to marketing techniques (23). Advertisements presented to children predominantly promote processed foods (24,25), including advertisements shown on YouTube (26). Another systematic review identified a range of food marketing techniques, with “television/movies” being the most studied vehicle in this respect; data showed that marketing influenced children’s consumption and attitude toward the advertised foods (21). However, the authors discussed the difference between implicit and explicit messages of food marketing as an area of future research, to enhance understanding of its impact. Our study found that UPF were eaten more frequently than foods of other NOVA categories, which seems to be closer to an explicit message for consumption; namely, eating the food is a stronger cue for modeling the food behavior than mere food presentation is, according to the social theory of learning (27).

Regarding the presence of healthier foods in the relevant literature, even with other than the NOVA classification systems, this is disappointing. Television series show a minimal representation of less-processed foods, including dairy, cheese, eggs, fruits, and vegetables (5,20). More particularly, Anderson and Anderson (7) found that television rarely showcased vegetables, ranking them as the second least-presented food item, while fruits primarily served decorative purposes rather than being depicted as nutritious sources of energy. Vegetables are minimally represented in children’s programming, particularly in shows created for tweens, as highlighted by Eisenberg et al (2). However, findings of our study showed an equal presentation of MPF and UPF per episode, which may be attributed to a series of educational context. For example, *Dora the Explorer* appeared to contain healthier food choices, while UPF items were rarely featured and even less frequently consumed. A more pedagogical content in the *Dora* series was also noted by Angin (28). Also, some of the older series in our sample, such as *Tom and Jerry*, clearly featured less UPF and more MPF. Although it was not the focus of our study, we noted that there also seems to be a neutral perception of healthier foods, such as fruits, in children’s programming. While fruits are sometimes associated with neutral or unfavorable meanings (5), vegetables receive even less attention, typically in neutral contexts (5,17). Additionally, Oates and Newman (29) noted that fruits, despite their visibility, were often used as background elements or props in children’s television shows. Our analysis also found that MPF items such as fruits and vegetables were primarily shown on screen but were not typically included in actual eating scenes.

Regarding the differences in results based on the continent of production, our study showed that European cartoons had a greater number of UPF compared with North American cartoons. Few other studies have investigated the food-related content of children’s television programming in relation to its geographical origin. A study assessing the educational value of food placements in 250 children’s movies examined the potential variations in food messages across different continents (19). European series exhibited the highest frequency of food placements; however, North American series were more inclined to depict noneducational food messages — particularly those endorsing foods of low nutritional value, such as sweets or sugary products — in a positive light by the characters. On the other hand, the duration of educational placements promoting healthier foods was significantly longer in Asian series than in European or North American ones (19). Scully et al (30) compared data from children’s programming in UK and Irish television stations regarding the frequency and type of foods presented. Most of the selected television programs (89%) in both television stations were produced in the US, the UK, or Ireland. However, in their report, the researchers opted not to categorize them based on the country of production. Li et al (10) found that many food references in Chinese programs viewed by children concerned healthy products.

To the best of our knowledge, this is the first study examining food-related content in popular children’s media that considers the degree of food processing and geographic origin. Nevertheless, the contribution of the study could be clarified and strengthened for public health nutrition, acknowledging certain limitations. One important temporal limitation is that the series we examined were broadcasted exclusively between 2011 and 2012, which restricts the years of data coverage and means the data are more than a decade old. However, this is balanced by certain strengths, as these data reflect the state of television productions during those years and could well serve as a point of comparison for later periods. Also, even though it is conceivable that the content of series may have changed, many of these are still broadcast in their original form and continue to circulate widely via global syndication, streaming platforms, and cultural references. For example, the television series *Dora the Explorer* continues to be currently broadcast, despite the release of a new series featuring the protagonist as an adolescent. *Lazy Town* has been relicensed and aired globally, with recent deals including broadcasts in Latin America, French-speaking Canada, Eastern Europe, Slovenia, and Benelux (31). Another limitation is that the sample size was limited to a selection of series broadcasted on Greek television channels. The top 10 series in terms of audience viewership were purposefully chosen, but these may not fully represent the entire spectrum of available content. However, many of the cartoons examined herein enjoy universal acceptance, such as *Dora the Explorer*, *Tom and Jerry*, *SpongeBob SquarePants*, or *Penguins of Madagascar.* In addition, the NOVA system has some inherent limitations because debatable whether the degree of processing per se affects the nutritional value of a product is debatable (32), and some researchers believe that nutrient analysis is advantageous in this regard (33). Moreover, UPF products cannot always be easily distinguished. For example, industrial bread containing only wheat flour, water, salt, and yeast, is a PF, while if it contains emulsifiers or colors it is a UPF.

This study demonstrated the dominating presence of UPF in children’s television series. Moreover, it showed that UPF were more prevalent in the series produced in European countries compared with those from other continents. Since messages conveyed through television series may have negative consequences on children’s dietary choices in real life, it would be of benefit to the public to modulate the food content portrayed in television programs and create a media environment that fosters children’s health and well-being by promoting healthy stereotypes. In this respect, the collaboration of groups such as broadcasters, content creators, and regulatory bodies, along with enforcement and public awareness, could increase the effectiveness of public health programs created for children.

## Appendix

**Appendix Table:** Number of Foods in the Total Sample of Television Episodes Analyzed, by NOVA Food Category^a^ and by Continent of Country of Production

Category	Asia	Europe	North America
**Minimally processed foods**			
Foods eaten	8	9	31
Introduced visually or verbally	18	22	62
All foods featured	26	31	93
**Processed culinary ingredients**			
Foods eaten	0	0	2
Introduced visually or verbally	0	1	2
All foods featured	0	1	4
**Processed foods**			
Foods eaten	4	0	5
Introduced visually or verbally	0	0	15
All foods featured	4	0	20
**Ultraprocessed foods**			
Foods eaten	10	53	54
Introduced visually or verbally	12	58	91
All foods featured	22	111	145

^a^ Categories described in Monteiro et al (13).
